# Transient Viscosity Adjustment Using a Coaxial Nozzle for Electrospinning Nanofibers from Non-Spinnable Pure *m*-Poly(hydroxyamide)

**DOI:** 10.3390/polym16233414

**Published:** 2024-12-04

**Authors:** Yerim Kim, Jihwan Lim, Han Seong Kim, Jaejun Lee, Youngsang Chun, Dong-Hyun Cho, Chan Sol Kang, Sejin Choi

**Affiliations:** 1 School of Chemical Engineering, Pusan National University, Busan 46241, Republic of Korea; 2 Institute of Advanced Organic Materials, Pusan National University, Busan 46241, Republic of Korea; 3 Department of Polymer Science and Engineering, Pusan National University, Busan 46241, Republic of Korea; 4 Department of Advanced Materials Engineering, Shinhan University, 95 Hoam-ro, Uijeongbu-si 11644, Republic of Korea; 5 Department of Aerospace Engineering, Pusan National University, Busan 46241, Republic of Korea

**Keywords:** *m*-PHA, vapor pressure, evaporation, solidification, fiberization, sheath-core, spinnability, SEM, rheological characteristic

## Abstract

In this study, a transient viscosity adjustment method using a coaxial nozzle was explored to fabricate nanofibers from non-spinnable *m*-poly(hydroxyamide) (*m*-PHA). Unlike conventional electrospinning methods that often require additives to induce fiber formation, this approach relies on a sheath-core configuration, introducing tetrahydrofuran (THF) to the sheath to temporarily adjust solution viscosity. The diffusion of THF into the core *m*-PHA solution resulted in momentary solidification at the interface, promoting nanofiber formation without compromising polymer solubility. SEM and rheological analyses confirmed that optimized sheath-to-core flow ratios yielded nanofibers with significantly reduced particle formation. Notably, increasing the THF flow rate facilitated a faster solidification rate, enhancing jet elongation and resulting in uniform nanofibers with diameters of approximately 180–190 nm. Although complete nanofibers without beads were not achieved in this study, this coaxial electrospinning approach presents a possible pathway for fabricating nanofibers from polymers with limited spinnability, potentially expanding the application scope of electro-spun materials in high-performance fields.

## 1. Introduction

Poly(benzoxazole) (PBO) is a polymer consisting of repeating benzoxazole units and a heterocyclic derivative with aromatic rings that imparts exceptional mechanical properties and chemical resistance due to its molecular structure [1,2,3,4]. Additionally, PBO exhibits a low thermal decomposition rate and minimal smoke emission during combustion, which can be attributed to its high glass transition temperature and flame resistance, making it ideal for high-temperature applications [5]. With these outstanding mechanical and thermal characteristics, PBO is recognized as a high-performance material and is used in various industries, including high-temperature-resistant filters and membranes, protective materials, and specialized sectors such as the military and aerospace [6,7,8].

However, PBO has a rod-like molecular structure induced by conjugated double bonds and strong secondary bonding between aromatic rings. This rigid structure prevents PBO from melting and renders it insoluble in most organic solvents, except strong acids, making it particularly challenging to form PBO into different shapes [9,10]. Thus, alternative methods have been reported to first shape the polymer using *m*-poly(hydroxyamide) (*m*-PHA), a precursor containing phenolic hydroxyl groups in the polymer backbone, which provides moderate solubility in polar organic solvents, allowing it to be thermally converted to PBO through a cyclization reaction [11,12]. As an aromatic polyamide, *m*-PHA not only transforms into a structure resembling PBO through thermal cyclization but also offers high mechanical strength, thermal stability, and flame resistance, making it suitable on its own for industrial reinforcement materials and heat-resistant fibers [13].

Nanoscale polymer fibers are inherently flexible due to their dynamic degrees of freedom, and they possess a high specific surface area, a characteristic of three-dimensional nanostructures. These features provide them with differentiated physical and chemical properties compared to traditional bulk materials, making them highly promising for applications in membranes, electronic devices, and responsive materials [14,15,16,17,18,19]. Electrospinning is an efficient and accessible technique for fabricating nanofibers, relying on the electrostatic repulsion of a high-voltage-charged polymeric solution. This method accommodates a wide range of polymers soluble in appropriate solvents and has been further extended to melt-electrospinning, a process in which polymer melts are used instead of solutions [20,21,22,23].

The successful formation of nanofibers in electrospinning relies on the continuity of the produced fibers, which is determined by the elongation and solidification of the polymer solution as it is released from the nozzle and moves toward the metal collector [20,24]. To enable the continuous elongation of the polymer solution, the preceding polymer molecule needs to drag the next molecule through intermolecular interactions, specifically, the molecular entanglements [25]. Furthermore, the drag force among the entangled molecules must withstand the high drawing tension; otherwise, particle-like deposition may occur instead of nanofiber formation, suggesting that variations in the microstructural state of the polymer may lead to differences in spinnability [26]. 

To prevent particle formation and achieve uniform nanofibers, previous studies have often added other substances [27,28,29]. In particular, high-molecular-weight polymers have been introduced as matrix materials to enhance insufficient polymer entanglements. While this approach facilitates nanofiber formation, it still has the drawback of altering the inherent properties of the original material due to the characteristics of the additives.

Herein, we report an effective method for successfully fabricating nanofibers from a non-spinnable polymer. In conventional electrospinning systems, pure *m*-PHA forms submicron particles rather than continuous fibers due to the insufficient intermolecular interactions. Although increasing the solution concentration is a straightforward way to raise a polymer solution’s viscosity, *m*-PHA still exhibits inadequate entanglement to withstand drawing tension, even at higher concentrations. Moreover, overly concentrated solutions become oversaturated, which prevents a stable discharge from the nozzle. Thus, for pure *m*-PHA to be electro-spun without additives, the polymer solution must be stably released at the nozzle, drawn into the fine fibers, and solidified through a momentary increase in viscosity before splitting into particles. In this study, the coaxial nozzle was introduced to achieve a transient viscosity adjustment of the polymer solution, thereby enhancing spinnability through electro-spun-jet solidification prior to breakage. As a result, nanofibers were successfully fabricated using only *m*-PHA dissolved in a typical organic solvent.

## 2. Materials and Methods

### 2.1. m-PHA Synthesis

*m*-PHA was synthesized through a low-temperature solution polycondensation reaction using 3,3′-dihydroxybenzidine (DHB, 98.0%, Tokyo Chemical Industry, Tokyo, Japan) and isophthaloyl chloride (IPC, 99.0%, Sigma-Aldrich Inc., Saint Louis, MO, USA) as monomers, with dimethylacetamide (DMAc, anhydrous, 99.8%, Sigma-Aldrich Inc., Saint Louis, MO, USA) as the solvent. DHB (0.01 mol) and DMAc (30 mL) were added to a 100 mL three-neck flask and stirred with a magnetic stirrer. IPC (0.01 mol) was then gradually added. The reaction mixture of DHB and IPC, dissolved in DMAc, was stirred slowly in an ice water bath (2 °C) under a nitrogen atmosphere for 1 h. The ice water bath was then removed, and the mixture was stirred at room temperature for an additional 20 h to complete the polymerization [13]. The intrinsic viscosity of the synthesized *m*-PHA was measured using an Ubbelohde viscometer, and a value of 1.5 dL g^−1^ was obtained.

### 2.2. Polymer Solution Preparation

The organic solvents used to examine the solubility of the *m*-PHA were DMAc, THF (anhydrous, 99.9%, Sigma-Aldrich Inc., Saint Louis, MO, USA), acetone (99.5%, DUKSAN Co., Seoul, Republic of Korea), acetonitrile (99.5%, DUKSAN Co., Seoul, Republic of Korea), and chloroform (99.8%, Fluka Co., Charlotte, NC, USA). Electrospinning solutions were prepared by dissolving the polymer at various concentrations in weight percent (wt%). The designations of the polymer solutions, based on solvent and concentration, are presented in Table 1. To improve solubility, lithium chloride (LiCl, anhydrous, DUKSAN Co., Seoul, Republic of Korea) was added at 10 wt% relative to the polymer in each solution and subsequently dissolved gradually over 12 h at 60 °C.

### 2.3. Electrospinning the m-PHA

**Figure 1:**
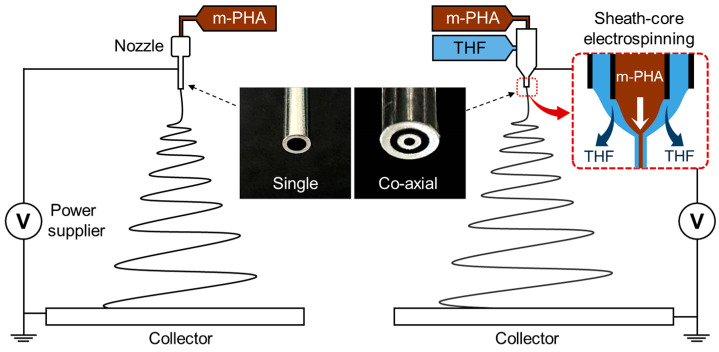
Schematic of the electrospinning setup using single and coaxial nozzles.

Electrospinning was performed using a high-voltage power supply (NanoNC Co., Seoul, Republic of Korea). Different voltages were applied to the nozzle, enabling vertical electrospinning directed towards an aluminum round collector positioned below. These voltages were optimized to ensure the stable release of the polymer solutions across various concentrations. Electrospinning was conducted in the following two configurations: one with a single metal nozzle (25 gauge, inner diameter: 0.26 mm) and the other with a sheath-core coaxial nozzle (Figure 1). The solution was fed into the single nozzle at a flow rate of 0.5 mL h^−1^ using a syringe pump (NanoNC Co., Seoul, Republic of Korea). Similarly, the solution was consistently supplied to the core part of the coaxial nozzle (inner diameter: 0.25 mm) at the same flow rate, while THF was delivered through the sheath (thickness: 0.18 mm) at variable flow rates. The nozzle tip-to-collector distance (TCD) was fixed at 170 mm. The detailed conditions for each setup are also presented in Table 1. All electrospinning processes were conducted at room temperature and 25% RH, with each product dried at room temperature for 24 h to remove the residual solvents.

### 2.4. Dynamic Behavior of the Electrospinning

**Figure 2:**
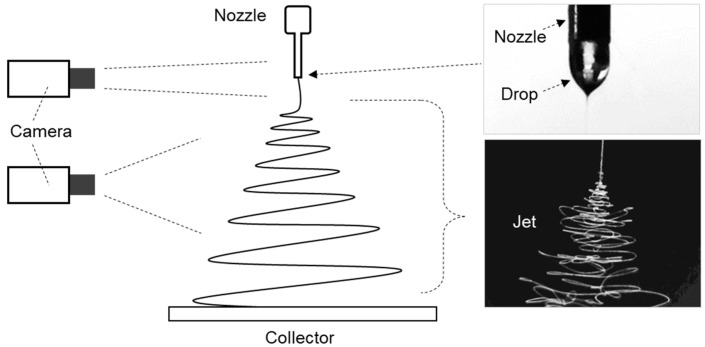
Schematic of the image capturing system for monitoring the drop and jet during electrospinning.

The liquid droplet released from a nozzle tip is referred to as a drop, while the liquid fiber ejected from a charged droplet by applied high voltage is termed a jet. The electro-spun drop and jet were continuously captured by a digital camera (HY-500D, Shenzhen Hayear Electronics Co. Ltd., Shenzhen, China) throughout the spinning process, as shown in Figure 2.

### 2.5. Rheological Characteristics

A rheometer (DHR 1, TA Instruments Co., New Castle, DE, USA) was employed to measure the viscosity of the *m*-PHA solution under the varying solution compositions.

### 2.6. Morphological Characteristics

The resultant electro-spun products were examined by scanning electron microscopy (SNE-4500M, SEC Co. Ltd., Suwon, Republic of Korea) to observe the morphological characteristics. The diameters and the distributions of the products were measured using a custom-developed image analysis program, which converted the image pixels to actual scales.

## 3. Results and Discussion

### 3.1. Solubility of the m-PHA in the Organic Solvents

**Figure 3:**
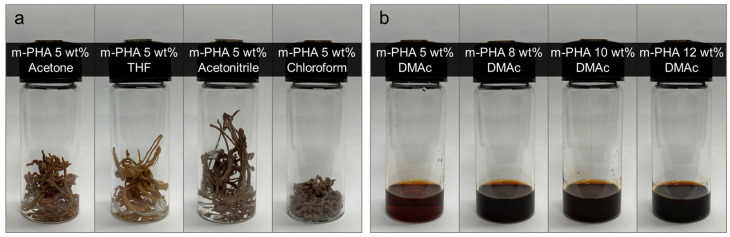
Solubility of the *m*-PHA in various organic solvents: (**a**) the insoluble solvents, and (**b**) soluble in DMAc at various concentrations.

To determine suitable solvents for dissolving the *m*-PHA, the solubility of the polymer in various organic solvents was examined. The primary considerations for electrospinning suitability included the dielectric constant and relative polarity to support charged jet formation, viscosity to withstand the strong tensile forces during jet elongation, and the boiling point for rapid solidification of the jet. Based on these factors, acetone, THF, acetonitrile, chloroform, and DMAc were investigated as candidate solvents. All solutions were prepared at concentrations of 5 wt%, with LiCl added to improve solubility. As a result, the *m*-PHA was completely insoluble in acetone, THF, acetonitrile, and chloroform, as shown in Figure 3a. In contrast, Figure 3b demonstrates that the *m*-PHA was soluble in DMAc, achieving complete dissolution at concentrations of up to 12 wt%.

### 3.2. Effect of the Solution Properties on Nanofiber Formation

The success of nanofiber fabrication is generally verified by examining the morphology of electro-spun products using a microscope or SEM. Thin, smooth, submicron fibers indicate that a polymer solution, charged by high voltage, underwent uniform elongation through whipping behavior. In contrast, when the drag force between molecular entanglements is insufficient to counter the tensile force acting on an emitted jet, the unsolidified jet breaks into particles. While this approach provides direct evidence of spinnability, the considerable time gap between observing the product morphology and adjusting the process parameters limits an immediate cause-and-effect analysis.

The stable fabrication of nanofibers depends on the dynamic behavior of the polymer solution as it travels from the nozzle to the collector. The behavior of the charged solution under high voltage is represented by the movement of the drop and jet—namely, the solution droplet dangling at the nozzle tip and the drawn fibers emitted from the droplet. Thus, observing the behavior of the drop and jet provides the visible evidence essential for successful nanofiber fabrication, enabling immediate and precise process control.

**Figure 4:**
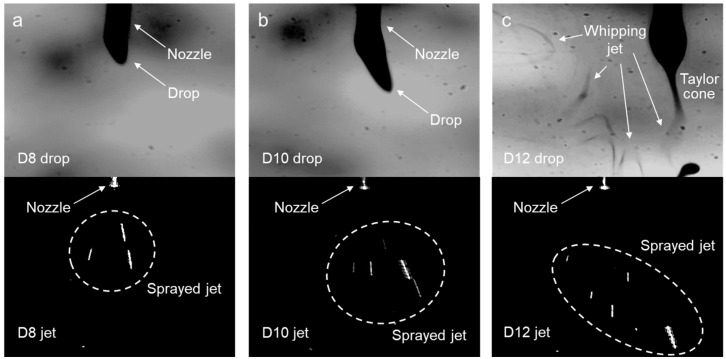
Drop and jet images of the electro-spun *m*-PHA solutions at the different concentrations in DMAc: (**a**) 8 wt%, (**b**) 10 wt%, and (**c**) 12 wt%.

The dynamic behavior of the electrospinning was examined using well-prepared polymer solutions at concentrations of 8, 10, and 12 wt% (referred to as D8, D10, and D12, respectively). Notably, the *m*-PHA solutions with concentrations exceeding 12 wt% were oversaturated and could not fully dissolve. The drop and jet images of D8 are shown in Figure 4a, where a sprayed jet was observed rather than a fiber-shaped jet exhibiting whipping behavior. Whipping is a key process in nanofiber formation, wherein a high charge density in the jet causes rapid, random elongation [30]. At this stage, molecular entanglements should pull together to form continuous fibers. However, the insufficient molecular network in D8 resulted in jet breakage. D10, which was expected to have a higher degree of entanglement than D8, allowed for some elongation of the solution due to electrostatic repulsion, leading to a slightly longer drop, as shown in Figure 4b. Nevertheless, a sprayed jet was still observed instead of continuous spinning, as the molecular structure remained insufficient for fiber formation. Interestingly, despite the sprayed jet observed in D12, a whipping jet was discovered near the drop, as shown in Figure 4c. Furthermore, a concave Taylor cone, attributed to the elongational flow of the solution, was distinctly visible. The increased molecular entanglement in D12, owing to its higher concentration, appeared sufficient to initiate whipping behavior. However, this entanglement appeared inadequate to withstand the high tensile forces, ultimately resulting in jet breakage.

**Figure 5:**
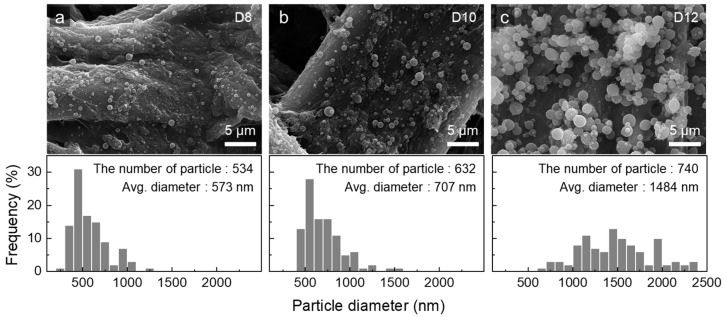
SEM images and diameter distributions of the particles produced from the *m*-PHA in DMAc solutions at concentrations of (**a**) 8 wt%, (**b**) 10 wt%, and (**c**) 12 wt%.

The electro-spun products from D8, D10, and D12 were obtained in forms corresponding to the drop and jet behaviors of each solution. Sprayed jets tend to solidify into spherical particles due to the surface tension of the solution during electrospinning as it travels toward the collector. The deposition of D8, which had the lowest viscosity, resulted in very small particles with an average diameter of 573 nm, as shown in Figure 5a. With a slightly higher elongation viscosity, D10 formed particles averaging 707 nm in diameter (Figure 5b). D12 produced the largest particles, averaging 1484 nm in diameter, owing to its more developed molecular network (Figure 5c). As the absolute amount of dissolved polymer increased, the number of generated particles also increased. Although additional entanglements tend to enhance intermolecular cohesion, no fiber-like morphology was observed in any of the samples. The absence of any stretched forms, such as deformed or oval-shaped particles, further suggests that factors beyond weak intermolecular cohesion could have been inhibiting elongation.

As mentioned above, producing a homogeneous, high-concentration solution with a well-developed polymer network to enable jet elongation was difficult to achieve. Moreover, the perfectly spherical shapes of the collected particles suggested that the sprayed jet had inordinately more than sufficient time to solidify into spheres. The slow evaporation rate of the solution did not support fiber formation. Thus, a mixed solvent of DMAc and THF was introduced to enhance evaporation.

**Figure 6:**
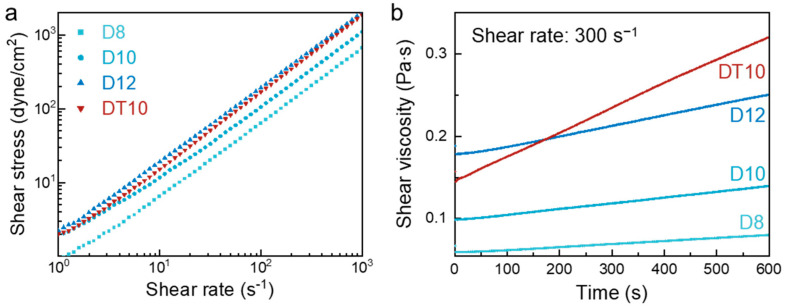
Rheological characteristics of the *m*-PHA solutions with various compositions: (**a**) shear stress change with shear rate, and (**b**) viscosity change over time at a fixed shear rate of 300 s^−1^.

Figure 6a compares the rheological properties of the solutions with the different compositions. THF was selected for its rapid volatility and miscibility with DMAc; however, as a non-solvent for *m*-PHA, it restricted the solution concentration to below 10 wt% (referred to as DT10). D8, D10, and D12 displayed increased shear stress with the concentrations, as increased molecular entanglements demand higher stress for shear deformation. Interestingly, DT10, prepared with a mixed solvent, initially began at a shear stress level similar to D10 but shifted to be closer to D12 as the shear rate increased. Since a different tendency was observed between D10 and DT10, which had the same polymer composition, this was concluded to have been influenced by the elapsed time rather than the changes in molecular structure. Figure 6b presents the change in solution viscosity over time. The viscosity of DT10 exhibited a more dramatic change over time compared to the single-solvent solutions, demonstrating that the rapid evaporation rate of THF enabled the effective control of the polymer solution viscosity. Moreover, the viscosity reversal between DT10 and D12 at 172 s suggested that the DMAc-THF mixture could effectively address the excessively prolonged solidification time observed during the stretching of the D12 jet.

**Figure 7:**
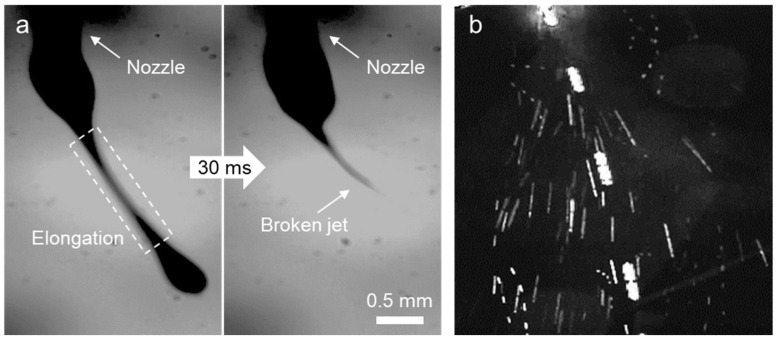
Images of the (**a**) drop and (**b**) jet of the electro-spun *m*-PHA solution at a 10 wt% in a DMAc-THF mixed solvent.

The sequential behavior of the drop and corresponding jet of DT10 was investigated. After being released from the nozzle, DT10 was expected to rapidly increase in viscosity, allowing it to withstand the high tensile force exerted on the jet. However, as shown in Figure 7a, the jet emitted from the drop extended momentarily but then broke before sustaining elongation. The image of the jet in Figure 7b further illustrates the sprayed jet, clearly highlighting this behavior. While DT10 showed improved electrospinning behavior compared to D10 with the same polymer composition, the evaporation rate still appeared to be too slow to achieve satisfactory jet elongation.

**Figure 8:**
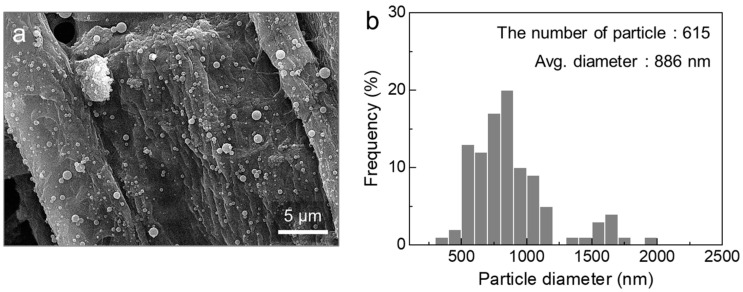
(**a**) SEM image and (**b**) diameter distribution of the particles obtained from the *m*-PHA solution at 10 wt% in a DMAc-THF mixed solvent.

The micro-particles formed from DT10, as observed in the SEM images, reflected similar findings (Figure 8a). Notably, the diameter distribution and number of particles produced from DT10 were slightly greater than those produced from D10 (Figure 8b). The morphological characteristics of the DT10 particles, which were positioned between those of D10 and D12, indicated that the faster solvent evaporation rate effectively enhanced the jet elongation. Nevertheless, achieving continuous fiber formation required an approach beyond simply adjusting solution properties, necessitating a fresh perspective and novel strategies.

### 3.3. Modifications to the Electrospinning Setup

Due to the solubility limits of the m-PHA, it was difficult to further increase the solution viscosity by raising concentration. Additionally, while controlling the solidification rate via a mixed solvent demonstrated promise, we found that it was ultimately insufficient, as the required evaporation rate exceeded that achievable by the solvent mixture. Although further addition of THF may have enhanced this approach, solubility remained a challenge. This exploration, however, suggested an alternative method: modifications to the electrospinning setup through the introduction of a coaxial nozzle. A coaxial nozzle is primarily used to create fibers composed of different materials in a sheath and core. We focused on the sheath part, where an additional substance was injected, and tried to induce a momentary viscosity change in the jet by flowing THF along this layer.

**Figure 9:**
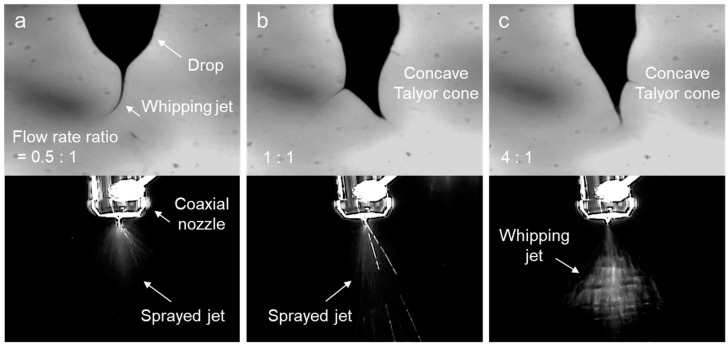
Drop and jet images of the electrospinning D10 using a coaxial nozzle at different sheath-to-core flow ratios: (**a**) 0.5:1, (**b**) 1:1, and (**c**) 4:1.

THF flowing from the sheath initiates a material exchange with a core solution, based on the diffusion principle from the concentration gradient [31]. Consequently, while the polymer solution in contact with THF momentarily solidifies at the interface, the volatile THF only transiently increases solution viscosity, thereby avoiding any reduction in *m*-PHA solubility. In other words, THF enables a jet to reach the necessary viscosity for elongation without compromising the solubility of a polymer. Thus, the optimal sheath flow rate to achieve this effect was investigated. Figure 9a shows images of the drop and jet when the THF flow rate in the sheath was 0.25 mL h^−1^, which was 0.5 times the fixed core flow of D10 (0.5 mL h^−1^). Similar to the electrospinning of the single-solvent D12 solution investigated earlier, the jet whipping of D10 was observed immediately after ejection from the drop. However, the viscosity remained insufficient to sustain continuous elongation, resulting in a sprayed jet again. At a slightly higher sheath flow rate (0.5 mL h^−1^), a transient increase in viscosity induced the formation of a concave Taylor cone, though it still did not prevent the jet from breaking into particles, as shown in Figure 9b. Continuous elongational flow of the *m*-PHA solution was initiated only when the sheath flow rate was increased to four times (2.0 mL h^−1^) that of the core. The concave Taylor cone induced by elongational flow was clearly visible, and the random, continuous whipping jet caused by electrostatic repulsion was evident, as shown in Figure 9c. We note that the white trail in the jet image of Figure 9c represents the afterimage of the rapidly moving whipping jet.

**Figure 10:**
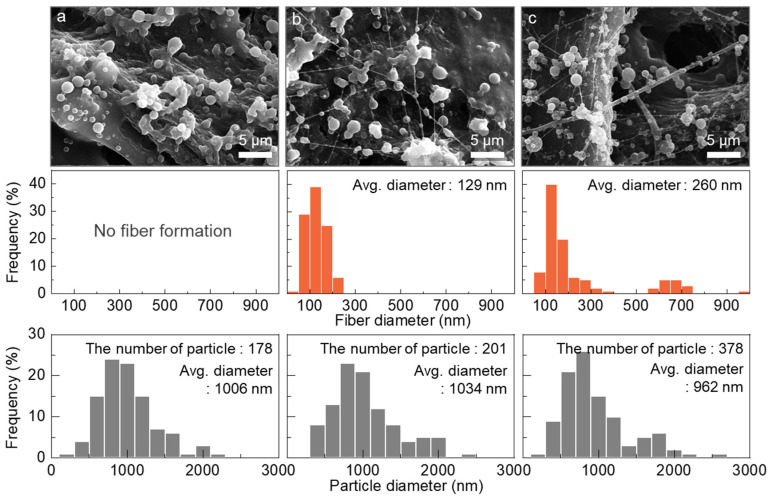
SEM images and diameter distributions of the nanofibers and particles produced from D10 using a coaxial nozzle at different sheath-to-core flow ratios: (**a**) 0.5:1, (**b**) 1:1, and (**c**) 4:1.

The SEM images also revealed the corresponding morphological characteristics. As shown in Figure 10a, stretched and deformed particles were produced at a flow rate ratio of 0.5:1, indicating that the solidification rate had become fast enough to fix the stretched form, which was not observed in either the single or mixed solvent electrospinning. At a flow rate ratio of 1:1, although in a small quantity, nanofibers with an average diameter of 129 nm were finally identified, as seen in Figure 10b. At a ratio of 4:1, thicker and more numerous nanofibers with an average diameter of 260 nm were produced as expected (Figure 10c). However, the predominant particle formation implied that there was still room to enhance intermolecular cohesion within the *m*-PHA solution.

**Figure 11:**
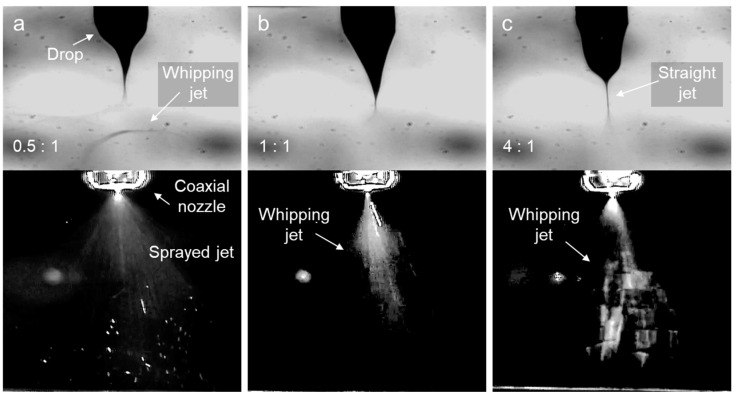
Drop and jet images of the electrospinning D12 using a coaxial nozzle at different sheath-to-core flow ratios: (**a**) 0.5:1, (**b**) 1:1, and (**c**) 4:1.

To further enhance intermolecular cohesion, the core solution was replaced with D12 instead of D10. Figure 11a shows that, similar to D10, jet whipping behavior occurred at a sheath flow rate that was half of the core flow rate. A notable difference from D10 was the formation of a concave Taylor cone, indicating more developed drag flow between the entangled molecules. As the sheath flow rate increased, whipping jets became more pronounced, as evidenced by the larger afterimages in Figure 11b. Additionally, the drop maintained a stable shape without oscillation, likely due to the uniform stress transmission within the solution. Notably, the straight jet shown in Figure 11c was a critical behavior that occurred when the molecular cohesion within the solution surpassed the electrostatic elongation force [32], and this is typically associated with stable electrospinning. Therefore, it was inferred that the nanofibers were well produced at a sheath-to-core flow ratio of 4:1.

**Figure 12:**
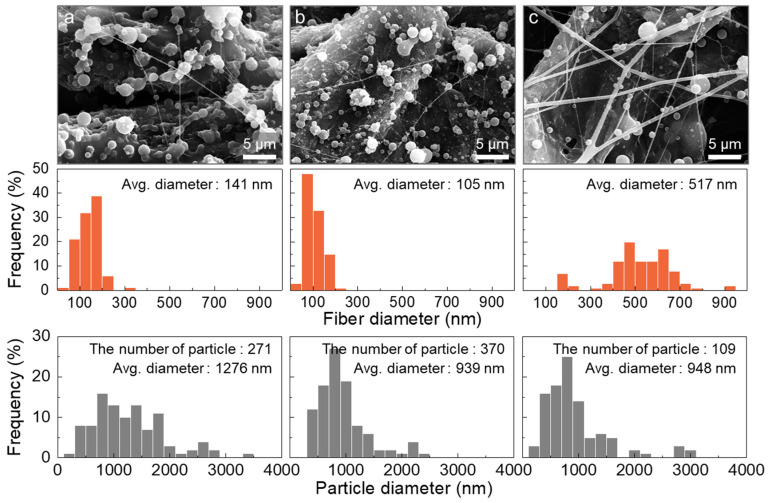
SEM images and diameter distributions of the nanofibers and particles produced from D12 using a coaxial nozzle at different sheath-to-core flow ratios: (**a**) 0.5:1, (**b**) 1:1, and (**c**) 4:1.

In line with expectations, the SEM images confirmed the partial formation of nanofibers from D12 under all flow rate conditions. At the lowest sheath-to-core flow ratio of 0.5:1, where the concave drop appeared, the collected morphology resembled that of D10 at a 1:1 flow rate (Figure 12a). This indicated that the increased polymer entanglement from the higher concentration of the polymer solution remained effective even with a coaxial nozzle setup. Although there was minimal difference at the 1:1 flow ratio of D12, which had a faster evaporation rate (Figure 12b), the highest ratio of 4:1, representing the fastest evaporation rate, yielded distinct nanofibers with an average diameter of 517 nm. Compared to the 4:1 flow ratio of D10, particle formation significantly decreased, as shown in Figure 12c. It was believed that the reductions in both particle count and size resulted from the fiber fabrication instead of the particles. Consequently, the control of evaporation rate through sheath flow adjustment was proven effective for the nanofiber formation. However, to further ensure the production of nanofibers, the THF flow rate in the sheath needed to be further increased to an extreme level.

**Figure 13:**
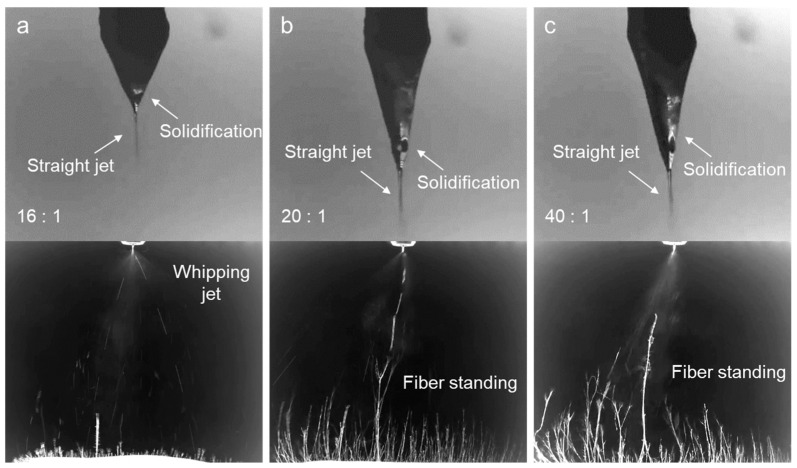
Drop and jet images of the electrospinning D12 using a coaxial nozzle at extremely high sheath-to-core flow ratios: (**a**) 16:1, (**b**) 20:1, and (**c**) 40:1.

To make nanofiber formation dominant over particle formation, the THF flow rate in the sheath was significantly increased to 8.0, 10.0, and 20.0 mL h^−1^, corresponding to sheath-to-core flow ratios of 16:1, 20:1, and 40:1, respectively. As the amount of THF contacting the *m*-PHA solution increased up to 16 times, solidification proceeded rapidly enough to be observable on the drop surface, as shown in Figure 13a. The appearance of a straight jet indicated behavior similar to typical electrospinning. When the THF flow rate was further increased by 20 times, providing an even faster evaporation rate, the drop became stretched further, as shown in Figure 13b. From this point, vertically standing fibers began to appear in the jet image, a phenomenon commonly observed when highly conductive polymer solutions are electro-spun. This behavior was likely due to the influence of the LiCl mixed with the *m*-PHA solution, indicating that nanofibers were being successfully formed at a 20:1 flow ratio. Similar electrospinning behavior was occurring at a 40:1 flow ratio, as shown in in Figure 13c, with standing fibers that appeared longer and more frequently. Under such extremely high sheath-to-core flow ratios, the dynamic behavior of electrospinning begins to closely resemble general electrospinning for very spinnable polymers.

**Figure 14:**
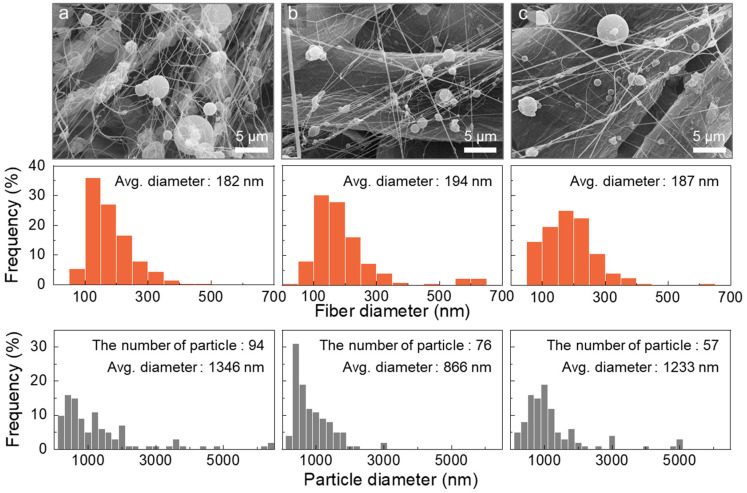
SEM images and diameter distributions of the nanofibers and particles produced from D12 using a coaxial nozzle at extremely high sheath-to-core flow ratios: (**a**) 16:1, (**b**) 20:1, and (**c**) 40:1.

In the SEM images of Figure 14, well-collected nanofibers with random structures are observed across all flow ratios. The fiber diameters were consistently measured approximately 180 to 190 nm, with minimal variations across the different flow ratios. Moreover, the number of particles gradually decreased from 94 to 76 and then to 57 as the flow ratio increased, indicating that fiber formation became increasingly dominant at higher flow ratios. The significant reduction in the number of micro-sized particles—from a maximum of 740 particles with a single nozzle to only 57 with the coaxial nozzle—was a remarkable achievement. Notably, this result was achieved solely through the modified nozzle design without any additional additives (Table 2). Although some particles remained mixed with the nanofibers and further improvement was needed, these findings suggested that fine-tuning the flow rate of the sheath solvent alone could potentially achieve complete nanofiber formation. Unfortunately, due to the maximum flow rate limit of the syringe pump used in this study, it was not possible to increase the sheath flow further. Nevertheless, we hope this report will serve as a valuable reference for future studies aimed at optimizing the electrospinning process.

## 4. Conclusions

The study successfully demonstrates the potential of a coaxial nozzle system for electrospinning nanofibers from pure *m*-PHA, a non-spinnable polymer in conventional setups. By introducing THF in the sheath, the coaxial configuration enabled transient viscosity adjustments, fostering the jet elongation and solidification essential for nanofiber formation. Rheological tests and SEM analysis showed that the appropriate sheath-to-core flow ratios reduced particle generation, producing nanofibers with uniform diameters and random structures. This result highlights that sheath flow modulation alone can enable nanofiber formation from challenging polymers like *m*-PHA without compromising the material’s inherent properties with additives. Future studies could explore the further optimization of flow rates and solvent compositions, offering insights into achieving fully uniform nanofiber structures in broader applications.

## Figures and Tables

**Table 1:** Electrospinning conditions with various nozzle types and solution compositions.

Nozzle Type	Solvent Type	Solution Concentration (wt%)	Solution Name	Applied Voltage (kV)	Flow Rate (mL h^−1^)		Others
					Core	Sheath	
Single (ID: 0.26 mm)	DMAc only	8	D8	10–11	0.5	-	Distance 170 mm
		10	D10				
		12	D12				
	DMAc:THF = 8:2 (*w/w*)	10	DT10	18	0.5	-	Room Temp
Coaxial (ID: 0.25 mm)	DMAc only	10	D10	22–24	0.5	0.25, 0.5, 2.0, 8.0, 10.0, and 20.0	25% RH
		12	D12				

**Table 2:** Comparison of similar studies.

Nozzle Type	Target Material	Additional Material	Ref.
Single nozzle	Low MW polymer	Gelatin	[33]
Single nozzle	High MW material	Low MW material	[34]
Single nozzle	Low MW polymer	Crosslinker	[35]
Coaxial nozzle	Ceramic	Polymer	[36]
Coaxial nozzle	Low MW polymer	None	This study

## Data Availability

The data presented in this study are available on request from the corresponding author.
